# Metformin improves lipid metabolism and reverses the Warburg effect in a canine model of chronic atrial fibrillation

**DOI:** 10.1186/s12872-020-01359-7

**Published:** 2020-02-03

**Authors:** Yaozhong Liu, Fan Bai, Na Liu, Baojian Zhang, Fen Qin, Tao Tu, Biao Li, Jiayi Li, Yingxu Ma, Feifan Ouyang, Qiming Liu

**Affiliations:** 1 Dept. of Cardiovascular Medicine/Cardiac Catheterization Lab. Second Xiangya Hospital, Central South University, No.139 Middle Renmin Road, Changsha, Hunan 410011 People’s Republic of China; 2 CCU department, affiliated hospital of traditional Chinese medicine, Xinjiang medical university, Urumqi, Xinjiang Province China; 3 Department of Cardiology, Asklepios-Klinik St Georg, Hamburg, Germany

**Keywords:** Atrial fibrillation, Metabolism, Metformin, Warburg effect, AMPK

## Abstract

**Background:**

Previous studies demonstrated impaired lipid metabolism and augmented aerobic glycolysis in AF. The authors aimed to investigate whether the use of metformin, an AMPK activator, could reverse this metabolic remodeling in chronic AF and to explore the underlying mechanisms.

**Methods:**

We conducted chronic AF animal models with 18 beagle dogs and divided them into SR (pacemaker implanted without pacing), AF (pacemaker implanted with sustained pacing at a frequency of 400 beats/min for 6 weeks), and metformin+AF group (daily oral administration of metformin was initiated 1 week before surgery and continued throughout the study period). After electrophysiological measurements, the left atrial appendage tissue samples were taken from the beating heart for further analysis. Protein expression, histological analysis, and biochemical measurements were conducted.

**Results:**

The AF groups showed decreased expression of FAT/CD36, CPT-1, VLCAD, increased concentration of free fatty acid and triglyceride, and increased lipid deposition. The activation of AMPK/PGC-1α/PPARα pathway was decreased. The key factors of the Warburg effect, including HIF-1α, GLUT-1, PDK1, HK, and LDH, increased in AF group compared to SR group. The expression of PDH decreased significantly, accompanied by increased atrial lactate production. The extent of fibrosis increased significantly in the left atrial appendage of AF group. dERP, ∑WOV, and AF inducibility increased while ERP decreased in AF group compared to SR group. The use of metformin attenuated all these changes effectively.

**Conclusions:**

Metformin improves lipid metabolism and reverses the Warburg effect in chronic AF via AMPK activation. It attenuates atrial electrical and structural remodeling.

## Background

Atrial fibrillation (AF) is the most common arrhythmia. Epidemiologic data suggest that 1% of the total population has AF, and the number of people affected is projected to grow dramatically and to more than double over the next 2~3 decades [1]. AF can be categorized as first diagnosed, paroxysmal, persistent, long-standing persistent, and permanent AF [2]. The treatment of AF involves an integrated therapy containing stroke prevention, rate control, and rhythm control. Despite its epidemiological importance and more than 100 years of basic and clinical research, the fundamental mechanisms are still poorly understood and the limited efficacy of current treatment options possibly results from the incomplete understanding of the pathophysiology of this complex heart rhythm disorder.

Electrical remodeling, structural remodeling, and autonomic remodeling have been demonstrated to play important roles in the pathogenesis of AF [1]. Each of these can result from cardiac disease conditions and promote the development of AF; AF in turn causes AF-promoting abnormalities within each of these areas, contributing to the progressive nature of the arrhythmia. Recent studies have documented substantial changes in energy metabolism in human and experimental AF, indicating the role of metabolic remodeling [3]. However, little is known regarding the underlying mechanisms and the impact of those changes upon the initiation/persistence of AF.

Impaired lipid metabolism and relatively augmented aerobic glycolysis have become the metabolic characteristics of the fibrillating atria [4]. This metabolic remodeling can contribute to the progression and development of AF vice versa, leading to the vicious cycle of ‘AF begets AF’. We have previously proposed that the decreased activation of AMP-activated protein kinase (AMPK) while increased activation of hypoxia-inducible factor 1α (HIF-1α) might be attributable to this metabolic pattern [4]. AMPK is an important sensor for cellular energy status. Once activated, it promotes fatty acid metabolism through peroxisome proliferator-activated receptor coactivator 1α (PGC-1α)/peroxisome proliferator-activated receptor coactivator 1α (PPAR-1α) pathway and reverses the energy shift from oxidative phosphorylation (OXPHOS) to aerobic glycolysis (the Warburg effect) via inhibiting HIF-1α. Previous studies have shown that alterations of AMPK are involved in AF pathogenesis [5], but how it regulates metabolism in AF remains unclear.

Metformin—an anti-diabetic drug, has been shown to possess protective effects on the cardiovascular system. Its widespread use has largely been supported by the United Kingdom Prospective Diabetes Study that reported lower cardiovascular mortality and morbidity in patients treated with metformin in comparison with alternative glucose-lowering drugs, despite similar glycemic control [6]. The MET-REMODEL trial proved that metformin treatment can significantly reduce left ventricular hypertrophy and oxidative stress in patients with coronary artery disease without diabetes [7]. Moreover, metformin has been demonstrated to inhibit the Warburg effect thereby exerting an anti-tumor effect [8]. Intriguingly, both the cardia-protective and anti-tumor role of metformin are predominantly attributed to its activation of AMPK. Therefore, we hypothesize that the use of metformin, an AMPK activator, could improve lipid metabolism and reverse the Warburg effect in AF.

## Methods

### Animal preparation

This study was performed in strict accordance with the recommendations in Guide for the Care and Use of Laboratory Animals of the National Institutes of Health [9]. The protocol was approved by the Committee governing the Ethics of Animal Experiments of the Wuhan University (SYXK(E)2004–0027). Eighteen male beagle dogs weighing 8–10 kg were obtained from the center of the experimental animal in the medical college of Wuhan University and bred with a standard diet. After the study, the dogs were sacrificed due to heart removal: dogs were anesthetized with 3% sodium pentobarbital and ventilated with a positive-pressure respirator (MAO01746; Harvard Apparatus, Holliston, MA). The initial dose was 1 ml/kg and an additional 2 ml/h was administered. Left and right-sided thoracotomies were performed at the fourth intercostal space. After electrophysiological measurements, the dogs’ hearts were cut off and the dogs die. All efforts were made to minimize suffering.

### Canine model of chronic AF

The canine chronic AF model was constructed by long-term rapid atrial pacing (RAP) as previously described [10]. A unipolar pacing lead was inserted into the right atrial appendage (RAA) under fluoroscopic guidance and connected to a pacemaker (AOO, Harbin University of Science and Technology, China) in the axillary pocket. The pacemaker was programmed to stimulate the right atrial (RA) at a frequency of 400 beats/min for 6 weeks. The success of this procedure was confirmed by electrocardiography. Sham-operated dogs were implanted with the same instrument but were maintained without pacemaker activation.

### Group setting

Eighteen beagle dogs were randomly assigned to three groups as follows:

(i) SR group (*n* = 6): sinus rhythm group, pacemaker implanted without pacing;
(ii) AF group (*n* = 6): pacemaker implanted with sustained pacing at a frequency of 400 beats/min for 6 weeks;
(iii) MET+AF group (n = 6): daily oral administration of metformin (100 mg/kg; Squibb Pharmaceutical, Shanghai, China) was initiated 1 week before surgery and continued throughout the study period.

### Electrophysiological measurements

Standard ECG limb leads were recorded at baseline and after 6-week RAP. Left and right-sided thoracotomies were performed at the fourth intercostal space. Multielectrode catheters were secured to allow pacing and recorded from the left and right atrial appendage (LAA and RAA), left and right atria (LA and RA). The electrophysiological parameters, including effective refractory period (ERP), ERP dispersion (dERP), window of vulnerability (WOV), and AF inducibility were measured as previously described [11]. Programmed stimulation of atrial myocardium was performed using the computer-based Lab System (Lead 7000; Jingjiang, Chengdu City, China)). ERP was determined by programmed pacing with 8 consecutive stimuli (S1-S1 = 300 ms) followed by a premature stimulus (S1-S2), which was progressively decreased until refractoriness was achieved. ERP dispersion was calculated offline as the coefficient of variation (standard deviation/mean) of ERP at all recording sites [12]. The difference between the longest and the shortest S1-S2 interval where AF was induced at each bipolar pair, was defined as WOV [12]. Cumulative WOV (∑WOV) was counted as the sum of WOVs from all sites in each dog. AF was defined as an irregular atrial rate faster than 500 beats/min associated with irregular atrioventricular conduction lasting > 5 s [13]. To determine AF inducibility, 10 consecutive bursts of RAP (cycle length 60 ms) at 4 sites for 2 s were implemented with 30s intervals. AF inducibility was calculated as the percentage ratio of AF number to total burst number.

### Tissue processing

After electrophysiological measurements, the left atrial appendage (LAA) tissue samples were taken from the beating heart. Then, the heart was cut off and the dogs died after experimentation. A part of LAA tissues was immediately frozen in liquid nitrogen to avoid changes in metabolic energy status, and the rest were fixed in 10% buffered neutral formalin for 24 h, routinely processed for paraffin embedding and cut into 5 mm thick serial sections used for chemical analysis for subsequent histochemical staining.

### Biochemical measurements

The lactate, free fatty acid (FFA), and the triglyceride (TG) content in LAA tissues were measured by kits from Nanjing Jiancheng Bioengineering Institute (Nanjing, China).

### Histological analysis

The extent of atrial fibrosis was detected by MASSON staining. The accumulation of lipid droplets in cardiac myocytes was detected by Oil Red O (AS1083, ASPEN) staining. Images were acquired using a Zeiss Imager DI microscope with a Zeiss AxioCam MRc5 color camera (Carl Zeiss, Oberkochen, Germany).

### Western blot analyses

Canine LAA tissues were lysed with RIPA Lysis Buffer (ASPEN, USA) supplemented with complete protease and phosphatase inhibitor cocktail (ASPEN, USA). Bicinchoninic acid (BCA) assay (ASPEN, USA) was used to estimated protein concentration after centrifugation at 13000 rpm for 5 min. Proteins were separated on SDS-polyacrylamide gels and transferred to PVDF membranes. Then, the membranes were incubated overnight at 4 °C with the following primary antibodies: anti-FAT/CD36 antibody (diluted 1:500, Bioss); anti-CPT-1 antibody (diluted 1:1000, Abcam); anti-VLCAD antibody (diluted 1:1000, Abcam); anti-GLUT1 antibody (diluted 1:500, Biorad); anti-HK antibody (diluted 1:500, Abcam); anti-PDK1 antibody (diluted 1:500, Biorbyt); anti-PDH antibody (diluted 1:500, Bioss); anti-LDH antibody (diluted 1:1000, Abcam); anti-AMPKα1 antibody (diluted 1:1000, Abcam); anti-p-AMPK (diluted 1:1000, Abcam); anti-HIF-1α antibody (diluted 1:1000, Thermofisher); anti-PGC-1α antibody (diluted 1:500, Abcam); anti-PPARα antibody (diluted 1:1500, Abcam); anti-β-actin antibody (diluted 1:10000, TDYbio); Secondary antibodies were goat anti-rabbit or anti-mouse, or rabbit anti-goat HRP-conjugated antibody (diluted 1:10000, ASPEN) for 30 min at room temperature. Antibody binding was detected with the ECL detection reagent (ASPEN, USA). Bands were quantified with Alpha Ease FC Software and results are shown as the ratio of total protein to β-actin normalized to control.

### Statistical analysis

Statistical analyses were performed using SPSS 22.0 software. All the values were expressed as mean ± SD. Kolmogorov-Smirnov tests and Levene’s test were used to test the normality of data distribution and variance equality respectively. The statistical significance of differences between the means was assessed by ANOVA and Tukey HSD for comparisons between two groups. A difference at *P* < 0.05 was considered statistically significant.

## Results

### Metformin alleviates AF-induced lipid accumulation in LAA

**Fig. 1:**
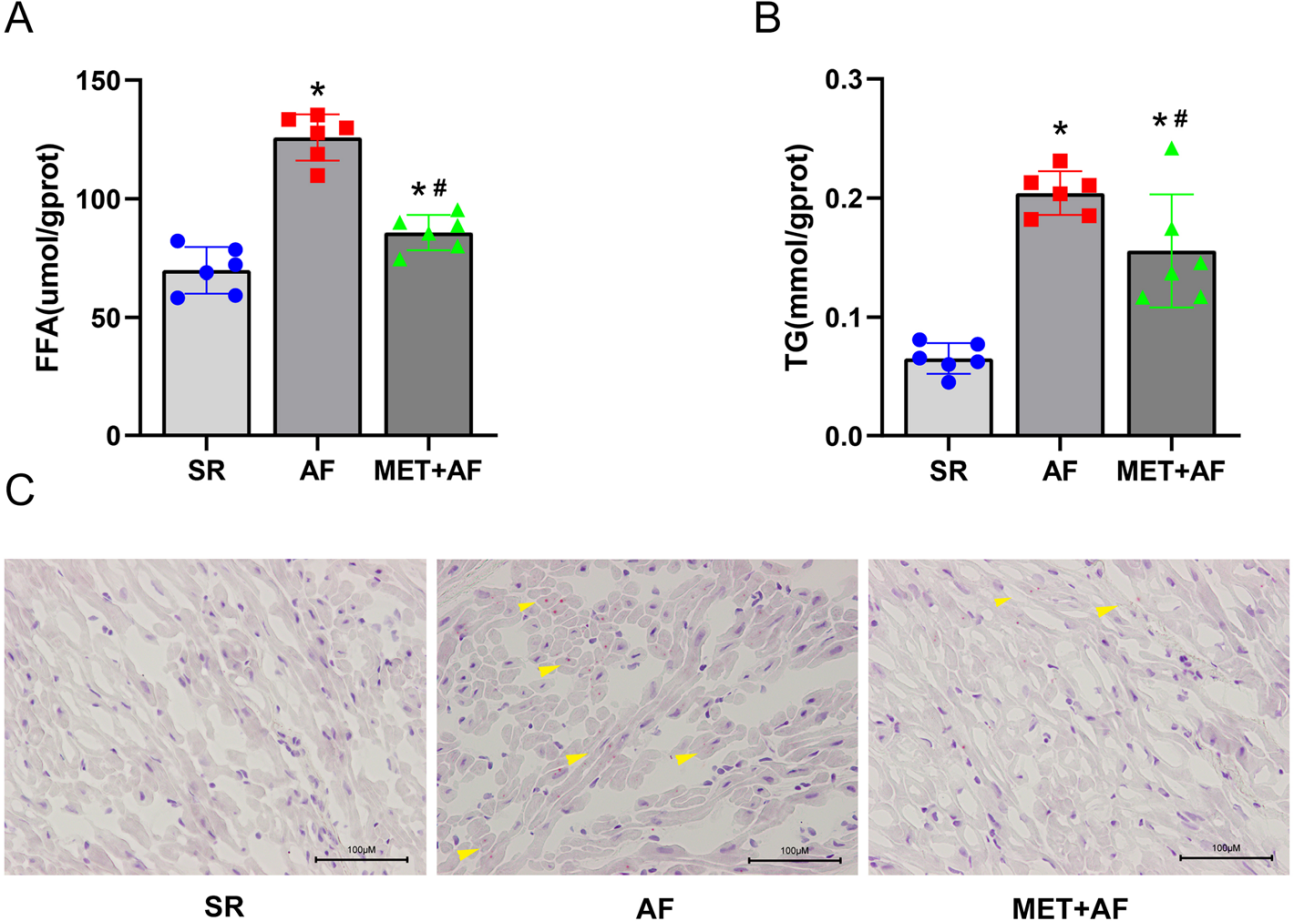
Metformin decreases lipid accumulation in chronic AF. **a**, **b** FFA and TG contents in LAA; **c** Representative images of oil red O staining for lipids (red color, × 40). SR, sinus rhythm; AF, atrial fibrillation; MET, metformin. * *P* < 0.05 versus SR group; # *P* < 0.05 versus AF group; *n* = 6 per group. (The yellow arrow points to the lipid droplets stained with red color)

Previous studies indicated impaired fatty acid metabolism and increased lipid accumulation during AF. We thought to determine whether the use of metformin could reverse these. The concentration of FFA (*FFA*, SR vs AF: 69.82 ± 9.76 vs 125.91 ± 9.81 umol/gprot, *P* < 0.01) and TG (*TG*, SR vs AF: 0.07 ± 0.01 vs 0.20 ± 0.02 mmol/gprot, *P* < 0.01) in LAA increased significantly at the AF group, both of which were alleviated by the use of metformin (*FFA*, AF vs MET+AF: 125.91 ± 9.81 vs 85.68 ± 7.50 umol/gprot, P < 0.01) (*TG*, AF vs MET+AF: 0.20 ± 0.02 vs 0.16 ± 0.05 mmol/gprot, *P* = 0.04) (Fig. 1a, b). Oil Red O staining revealed that AF group’s LAA tissues had large numbers of lipid droplets stained with a red color while metformin decreased the lipid contents (Fig. 1c). These data proved that metformin can decrease lipid accumulation in chronic AF.

### Metformin regulates protein expression of key fatty acid metabolic factors

**Fig. 2:**
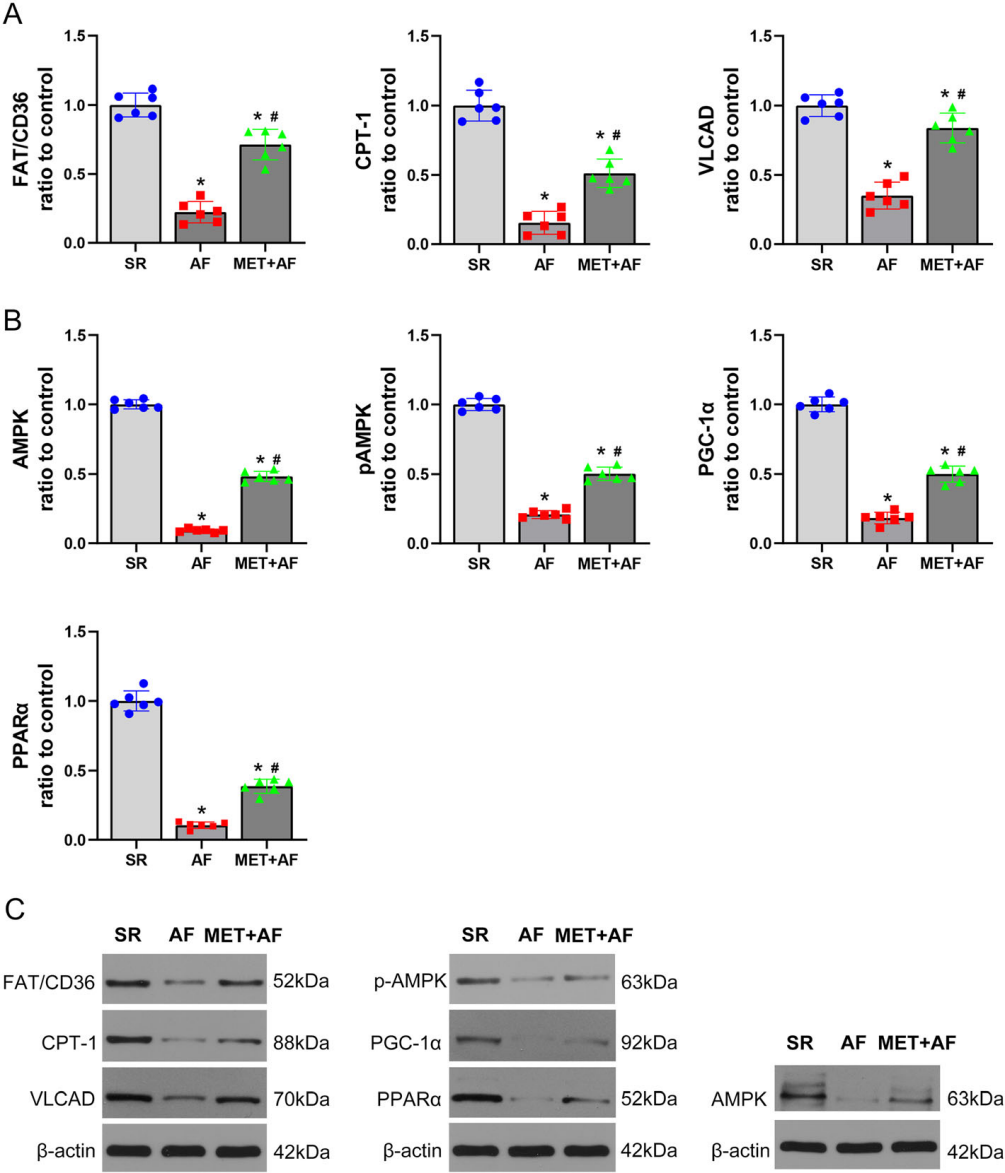
Metformin regulates the expression of key fatty acid metabolic factors in chronic AF. **a**, **b** Quantitative analysis of the protein expression of FAT/CD36, CPT-1, VLCAD (**a**) and AMPK, pAMPK, PPAR-α, PCG-1α (**b**) in three groups. **c** Representative images of the protein expression. SR, sinus rhythm; AF: atrial fibrillation; MET, metformin; FAT/CD36, fatty acid translocase; CPT-1, carnitine palmitoyl transferase-1; VLCAD, Very long-chain specific acyl-CoA dehydrogenase; AMPK, adenosine 5′-monophosphate (AMP)-activated protein kinase; pAMPK, phosphorylated AMPK; PPAR-α, peroxisome proliferator-activated receptor; PGC-1α, peroxisome proliferator-activated receptor-gamma coactivator 1α. * *P* < 0.05 versus SR group; # *P* < 0.05 versus AF group; *n* = 6 per group

To investigate how metformin regulates fatty acid metabolism, we examined the protein expression of key fatty acid metabolic factors in LAA from three groups, including fatty acid transporter (FAT/CD36), carnitine palmitoyl transferase-1 (CPT-1), and very-long-chain acyl-CoA dehydrogenase (VLCAD). As Fig. 2.a shown, all these factors were significantly down-regulated in AF group compared to SR group while the use of metformin restored them. As the AMPK/PGC-1α/PPARα pathway plays vital roles in regulating fatty acid metabolism by increasing the expression of these factors, we examined AMPK, phosphorylated AMPK (pAMPK), PPAR-α, and PGC-1α. This pathway was significantly downregulated during AF while the use of metformin increased its activation (Fig. 2b).

### Metformin reverses the Warburg effect in chronic AF

**Fig. 3:**
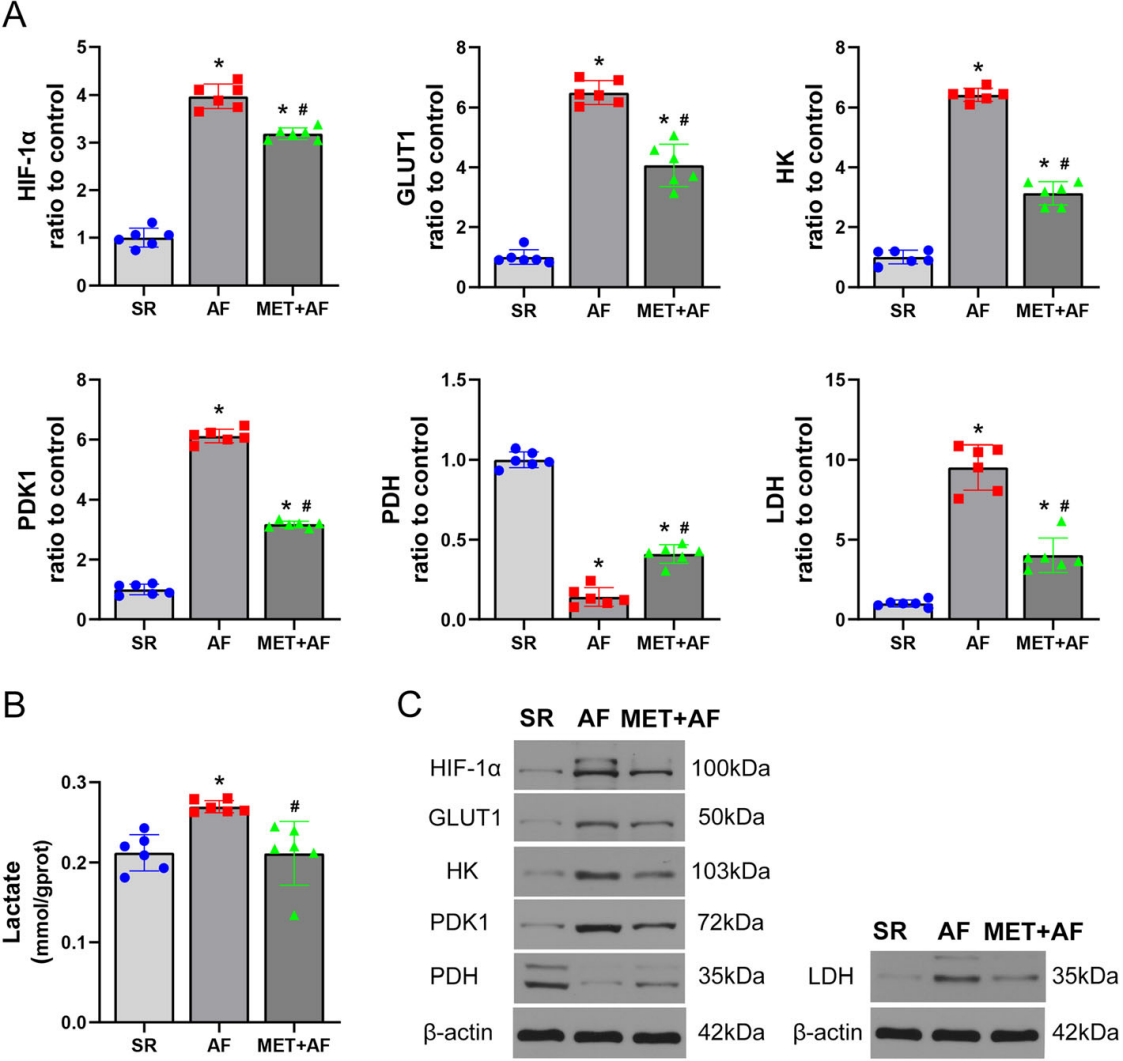
Metformin reverses the Warburg effect in chronic AF. **a** Quantitative analysis of key fators of the Warburg effect including HIF-1α, GLUT-1, PDK-1, PDH, HK, and LDH. **b** Analysis of the production of atrial lactate. **c** Representative images of the protein expression. SR, sinus rhythm; AF: atrial fibrillation; MET, metformin; HIF-1α, hypoxia-inducible factor 1α; GLUT-1, glucose transporter-1; PDK-1, pyruvate dehydrogenase kinase 1; PDH, pyruvate dehydrogenase; HK, hexokinase; LDH, lactate dehydrogenase. * *P* < 0.05 versus SR group; # *P* < 0.05 versus AF group; *n* = 6 per group

The Warburg effect is characterized by the augmented aerobic glycolysis, and the key factors in driving the Warburg effect include HIF-1α, glucose transporter-1 (GLUT-1), pyruvate dehydrogenase kinase 1 (PDK-1), hexokinase (HK), and lactate dehydrogenase (LDH). All these protein expressions were found significantly increased in AF group, accompanied by decreased pyruvate dehydrogenase (PDH) expression and increased lactate production (*Lactate*, SR vs AF: 0.21 ± 0.02 vs 0.27 ± 0.01 mmol/gprot, *P* < 0.01). The use of metformin reversed these changes (*Lactate*, AF vs MET+AF: 0.27 ± 0.01 vs 0.21 ± 0.04 mmol/gprot, *P* < 0.01) (Fig. 3).

### Metformin attenuates atrial electric remodeling and structural remodeling in chronic AF

**Fig. 4:**
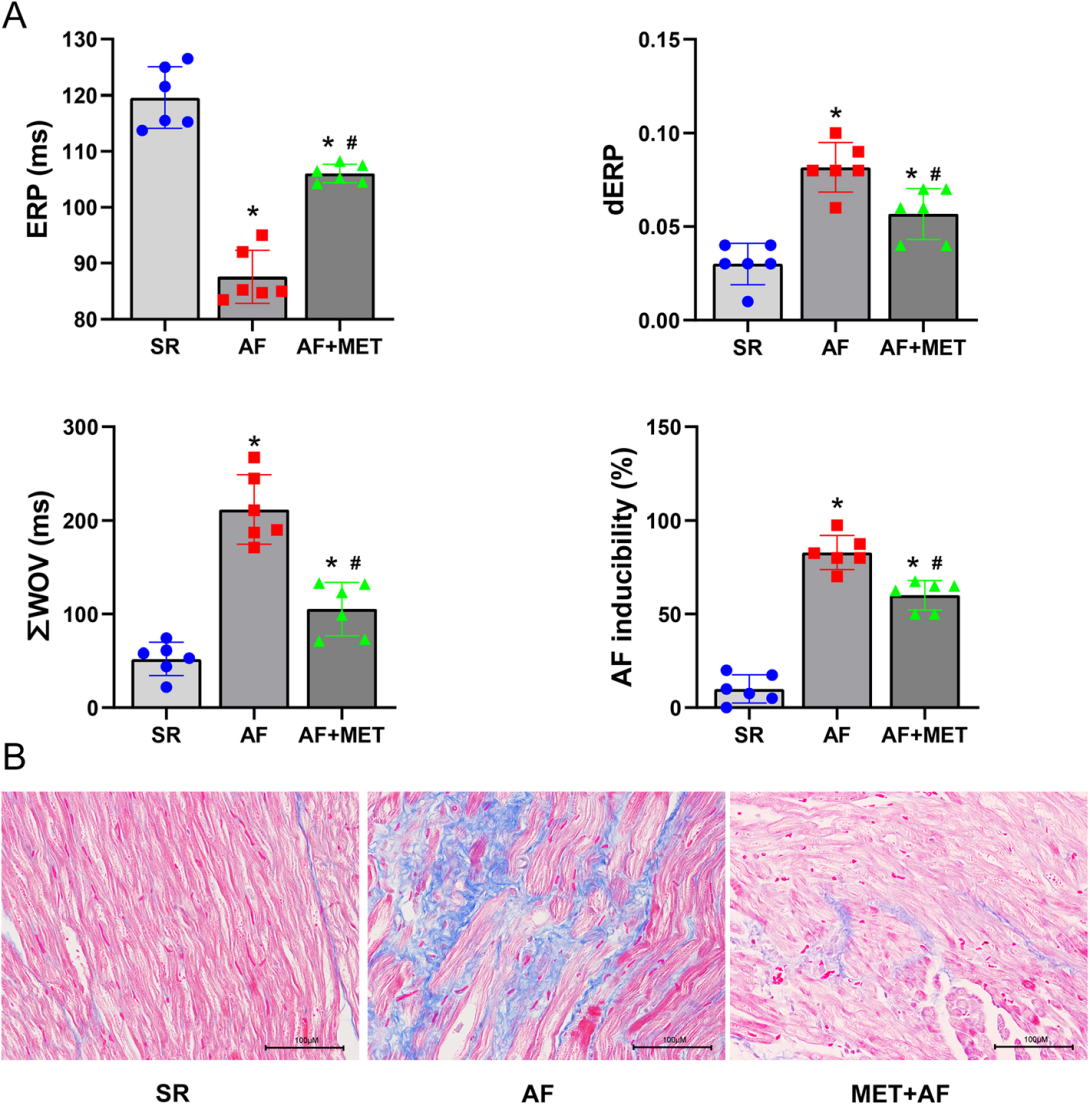
Metformin attenuates atrial electric remodeling and structural remodeling in chronic AF. **a** Analysis of ERP, dERP, ∑WOV, and AF inducibility. **b** Representative images of MASSON staining for atrial fibrosis (blue). ERP, effective refractory period; dERP, ERP dispersion; ∑WOV, cumulative window of vulnerability. * P < 0.05 versus SR group; # *P* < 0.05 versus AF group; *n* = 6 per group

Analysis of ERP, dERP, ∑WOV, and AF inducibility are shown in Fig. 4a. The AF inducibility (SR vs AF: 10.00 ± 7.58 vs 89.92 ± 9.14%, *p* < 0.01), ∑WOV (SR vs AF: 52.00 ± 17.70 vs 211.83 ± 37.16 ms, *p* < 0.01), and dERP (SR vs AF: 0.03 ± 0.01 vs 0.08 ± 0.01, *p* < 0.01) in AF group were significantly higher than those in SR group, while metformin treatment significantly reduced these (*AF inducibility,* AF vs MET+AF: 89.92 ± 9.14% vs 60.00 ± 7.91%, *p* < 0.01) (*∑WOV*, AF vs MET+AF: 211.83 ± 37.16 vs 105.17 ± 28.47 ms, *p* < 0.01) (*dERP*, AF vs MET+AF: 0.08 ± 0.01 vs 0.06 ± 0.01, *p* = 0.01). In contrast, the ERP in AF group was decreased (SR vs AF: 119.58 ± 5.48 vs 87.58 ± 4.72 ms, *P* < 0.01) and metformin treatment restored the ERP value (AF vs MET+AF: 87.58 ± 4.72 vs 106.04 ± 1.64 ms, *P* < 0.01).

Figure 4b shows the fibrosis extent of LAA in three groups. Masson staining revealed increased interstitial fibrosis in the LAA in AF group compared to that in SR group. Metformin significantly reduced atrial fibrosis. These data proved that metformin attenuates atrial electrical and structural remodeling in chronic AF.

## Discussion

The healthy heart relies predominantly (~ 60–90%) on fatty acid (FA) oxidation to fuel ATP production [14]. Circulating FAs enter cardiomyocytes via the FA transporter, FAT/CD36. CPT-1 then allows FA entry into mitochondria for β-oxidation. Our previous proteomics study showed that VLCAD, the initial rate-limiting enzyme in mitochondrial fatty acid β-oxidation, was decreased in the LAA tissue of permanent AF patients [15]. Previous studies also found decreased expression of CPT-1 in AF model [16]. These are consistent with the findings of our present study. In the chronic AF group, both the fatty acid uptake and oxidation were impaired, along with increased accumulation of lipids. This indicated decreased FA metabolism in AF.

PPARs and its coactivator, PGC-1α, play key roles in regulating heart fatty acid metabolism [17]. Activation of PPAR-α induces FA uptake and oxidation through upregulating the gene expression of FAT/CD36, CPT-1, VLCAD, etc. Previous studies demonstrated decreased activation of PGC-1α/PPARα pathway in chronic AF [16]. AMPK, which can improve fatty acids metabolism via phosphorylation of PGC-1α, was also found decreased in AF [5]. In the present study, we proved that the use of metformin, an AMPK activator, can upregulate the activation of PGC-1α/PPAR-α pathway, thereby increasing the expression of FAT/CD36, CPT-1, and VLCAD, and improving lipid metabolism.

When the lipid metabolism and OXPHOS are impaired, another way to produce ATP is through aerobic glycolysis, the so-called Warburg effect [18]. The Warburg effect is usually mentioned in relation to cancer cell growth, but recent studies begin to shed light on the importance of aerobic glycolysis in normal cells as an adaptive mechanism for minimizing oxidative stress. Previous studies have proved the existence of the Warburg effect in AF, as evidenced by the significantly increased atrial lactate production, up-regulated glycolytic enzyme, and down-regulated PDH complex [4]. But the specific mechanism remains unclear. HIF-1α plays important roles in regulating the Warburg effect. Hypoxia, mutation of VHL, or accumulation of reactive oxygen species (ROS) impair HIF-1α degradation, allowing it to enter the nucleus and engage in transcriptional activity. HIF-1α upregulate pyruvate dehydrogenase kinase (PDK) levels, thereby reducing PDH active levels. It also directly increase the expression of GLUT1, LDHA, and HK [19]. The combined effect on glucose metabolism is to increase both glucose utilization and lactate production. In tumors, AMPK has been demonstrated to down-regulate the expression of HIF-1α, thereby exerting ‘anti-Warburg’ [20]. The present study further proved the existence of the Warburg effect in AF and indicated that the reversal of the Warburg effect by metformin is mediated by HIF-1α inhibition via AMPK activation.

Intriguingly, the Warburg effect is also important to immune cell activation [18]. Inhibition of HIF-1α blocked monocyte induction, whereas the AMPK activator metformin inhibited the innate immune response [21]. Recent studies have begun to shed light on the immunometabolism regulator role of metformin in cardiovascular disease [6]. C. Sardu and his colleagues proved that the use of metformin can regulate the adipose tissue metabolism and improve the cardiac performance in pre-diabetic patients [22–25], and these effects are predominantly anti-inflammatory. Moreover, inflammation has long be regarded as a vital contributor to AF [26]. The infiltration of immune cells and proteins that mediate the inflammatory response in cardiac tissue and circulatory processes is closely associated with AF. Activated immune cells have been suggested to cause both electrical and structual remodeling in AF [27]. Therefore, it is rational to speculate that the role of metformin in AF may also partially due to its anti-inflammation role. Further studies are in need. There are several limitations in the current study: (i) the low number of animals in the three groups; (ii) the lack of cellular models to verify the possible mechanism; (iii) the lack of baseline data before metformin or placebo treatment, therefore we were only able to appreciate the values at the end of the experiment. Under these conditions, the collected data only has a cross-sectional disposition in time, which limits the possibility of extracting cause-effects deductions. (iiii) the inability to extrapolate conclusions to other species than male beagle dogs.

## Conclusion

The main findings of the present study are as follows: Firstly, metformin improved the impaired lipid metabolism in chronic AF, as manifested by the increased expression of FAT, CPT-1, VLCSD and decreased lipid accumulation in the MET+AF group compared with AF group. This effect may be mediated by the increased activation of AMPK/PGC-1α/PPAR-α pathway. Secondly, metformin inhibited the upregulation of key factors of the Warburg effect in chronic AF including HIF-1α, GLUT1, PDK1, HK, and LDH; increased the expression of PDH; and decreased the production of atrial lactate. Lastly, metformin attenuated atrial electrical remodeling and structural remodeling in chronic AF.

In summary, the use of metformin can improve lipid metabolism, reverse the Warburg effect, and decrease atrial remodeling in chronic AF. These findings emphasize the importance of AMPK in regulating metabolism in AF, provide new mechanistic insights, and point to new approaches and tools for therapeutic innovation.

## Data Availability

The datasets used and/or analysed during the current study are available from the corresponding author on reasonable request.

## Abbreviations

AF: Atrial fibrillation
AMPK: Adenosine 5′-monophosphate-activated protein kinase
CPT-1: Carnitine palmitoyl transferase-1
dERP: ERP dispersion
ERP: Effective refractory period
FA,: Fatty acid
FAT/CD36: Fatty acid translocase
FFA: Free fatty acid
GLUT-1: Glucose transporter-1
HIF-1α: Hypoxia inducible factor-1α
HK: Hexokinase
LAA: Left atrial appendage
LDH: Lactate dehydrogenase
MET: Metformin
OXPHOS: Oxidative phosphorylation
pAMPK: Phosphorylated AMPK
PDH: Pyruvate dehydrogenase
PDK-1: Pyruvate dehydrogenase kinase 1
PGC-1α: Peroxisome proliferator-activated receptor coactivator 1α
PPAR-α: Peroxisome proliferator-activated receptor α
ROS: Reactive oxygen species
SR: Sinus rhythm
TG: Triglyceride
VHL: Von Hippel-Lindau tumor suppressor
VLCAD: Very long-chain specific acyl-CoA dehydrogenase
WOV: Window of vulnerability
